# Genetic characterization of cassava (*Manihot esculenta*) landraces in Brazil assessed with simple sequence repeats

**DOI:** 10.1590/S1415-47572009005000010

**Published:** 2009-01-17

**Authors:** Marcos V. B. M. Siqueira, Jurema R. Queiroz-Silva, Eduardo A. Bressan, Aline Borges, Kayo J. C. Pereira, José G. Pinto, Elizabeth Ann Veasey

**Affiliations:** Departamento de Genética, Escola Superior de Agricultura Luiz de Queiroz, Universidade de São Paulo, Piracicaba, SPBrazil

**Keywords:** genetic diversity, microsatellites, SSR markers, traditional farming

## Abstract

Based on nine microsatellite loci, the aim of this study was to appraise the genetic diversity of 42 cassava (*Manihot esculenta*) landraces from selected regions in Brazil, and examine how this variety is distributed according to origin in several municipalities in the states of Minas Gerais, São Paulo, Mato Grosso do Sul, Amazonas and Mato Grosso. High diversity values were found among the five above-mentioned regions, with 3.3 alleles per locus on an average, a high percentage of polymorphic loci varying from 88.8% to 100%, an average of 0.265 for observed heterozygosity and 0.570 for gene diversity. Most genetic diversity was concentrated within the regions themselves (*H*_*S*_ = 0.52). Cluster analysis and principal component based scatter plotting showed greater similarity among landraces from São Paulo, Mato Grosso do Sul and Amazonas, whereas those from Minas Gerais were clustered into a sub-group within this group. The plants from Mato Grosso, mostly collected in the municipality of General Carneiro, provided the highest differentiation. The migration of human populations is one among the possible reasons for this closer resemblance or greater disparity among plants from the various regions.

## Introduction

Traditional or slash-and-burn farming is a system with characteristics related to the pre-colonial period, and which is preserved by both indigenous and other populations that employ techniques transmitted culturally by their ancestors (Faraldo *et al.*, 2000). The basic evolutionary unit of traditional farming, the “swidden field”, is where both *in situ* conservation of landraces from many species of economic importance and genetic amplification of diversity occur, with subsequent benefits to the farmer (Martins, 1994, 2001; Peroni and Martins, 2000; Sambatti *et al.*, 2001). The terms landrace, ethno-variety and folk or local variety define plant populations which are ecologically or geographically distinct, and are differentiated in their internal genetic composition, as a result of local selection by traditional farmers (Brown, 1978). Traditional agro-systems are of particular interest as they usually represent high crop diversity. It is common to find numerous varieties in the same field (Elias *et al.*, 2000). Some of these species have received attention by researchers through the focus on genetic characterization, for example sweet potato (Veasey *et al.*, 2007, 2008), yam (Malapa *et al.*, 2005), taro (Jianchu *et al.*, 2001), maize (Louette *et al.*, 1997), bananas (Creste *et al.*, 2003), and most important of all, cassava (Sambatti *et al.*, 2000; Mühlen *et al.*, 2000; Faraldo *et al.*, 2000; Peroni, 2007).

Cassava (*Manihot esculenta* Crantz), known in Brazil as “mandioca”, “macaxeira” or “aipim”, is the main crop cultivated in traditional farming systems in Brazil, as well as other areas in tropical America (Martins, 1994). It is an important subsistence crop for many communities with flexible planting and harvest times (Mkumbira *et al.*, 2003). Represented by a high number of varieties, cassava stands out as a suitable model for analyzing the inter-relationship between societies, genetic resources and ecological conditions. Studies concerning cassava diversity are scarce when compared with the great ethnical and territorial diversity of the populations that grow *M. esculenta*. Socio-cultural contexts, as well as economic and ecological processes, exert an influence on the management of this crop with variable intensity. The high diversity observed in those traditional populations that cultivate cassava reflects a pre- and post-colonial history, consisting of migrations, inter-ethnic contacts and economic pressures (Emperaire *et al.*, 2001).

In order to understand the important role of traditional farmers in maintaining and even amplifying genetic diversity in cassava landraces in Brazil and other countries, various studies have been undertaken with isozyme markers (Sambatti *et al.*, 2000; Faraldo *et al.*, 2000; Cabral *et al.*, 2002; Resende *et al.*, 2004), randomly amplified polymorphic DNA (RAPD) markers (Colombo *et al.*, 1998; Carvalho and Schaal, 2001; Zacarias *et al.*, 2004), and microsatellites or simple sequence repeats (SSR) (Mühlen *et al.*, 2000; Carvalho and Schaal, 2001; Peroni, 2007), the latter being an appropriate marker for the detection of genetic polymorphisms, widely used to characterize genetic diversity in traditional crops (Mühlen *et al.*, 2000; Fregene *et al.*, 2003; Elias *et al.*, 2004; Veasey *et al.*, 2008).

In order to assess the genetic diversity of local varieties in farmers' homesteads and their distribution throughout different regions in Brazil, as a means of devising better conservation approaches and identifying progenitors with a wide genetic base for breeding, 42 landraces were evaluated with nine SSR markers. The plants were divided into five groups according to geographic origin: MG - Minas Gerais; SP - São Paulo; MS - Mato Grosso do Sul; AM - Amazonas; and MT - Mato Grosso. Certain groups, such as MS and MT, were either not represented or poorly so in the previous studies mentioned above. Thus, further information should be propitious concerning the genetic diversity of cassava in these regions.

**Figure 1:**
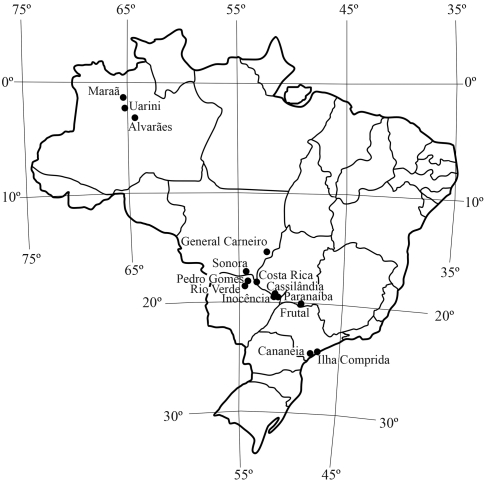
Map of Brazil showing the municipalities sampled in this study.

**Figure 2:**
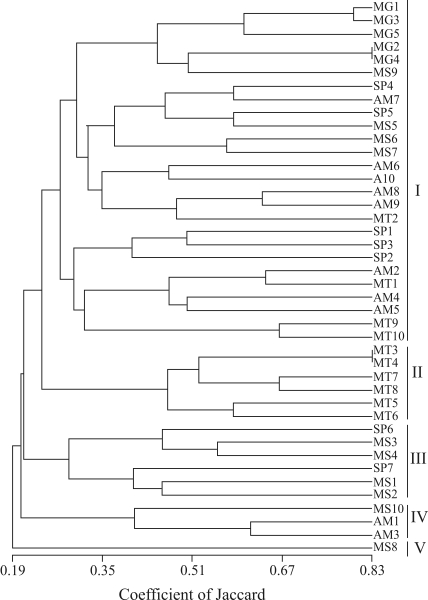
Dendrogram obtained with the Jaccard similarity coefficient and UPGMA method, representing genetic relationships among 42 landraces of cassava (*Manihot esculenta*): MG1 to MG5 from Minas Gerais (group MG), SP1 to SP7 from São Paulo (group SP), MS1 to MS10 from Mato Grosso do Sul (group MS), AM1 to AM10 from the Amazon (group AM), and MT1 to MT10 from Mato Grosso (group MT).

## Materials and Methods

### Plant material

A total of 42 landraces from the cassava germplasm bank of the Genetics Department of ESALQ/USP, Piracicaba, SP, were assessed according to their geographical origin, with the aim of obtaining a representative sample from different parts of Brazil. These plants were collected from homesteads undertaking traditional farming methods, in several municipalities in five regions of Brazil, and were classified into five groups, according to their place of origin: MG - municipality of Frutal, Minas Gerais State; SP - municipalities of Eldorado, Cananéia and Ilha Comprida, in the Vale do Ribeira, São Paulo State; MS - municipalities of Sonora, Pedro Gomes, Rio Verde de Mato Grosso, Costa Rica, Cassilândia, Paranaíba and Inocência, Mato Grosso do Sul State; AM - municipalities of Uarini, Maraã and Alvarães, in the Mamirauá and Amanã Sustainable Development Reserves, Amazonas State; and MT - municipality of General Carneiro, Mato Grosso State (Figure 1; Table 1).

### DNA extraction and quantification

DNA was extracted from recently expanded young leaves of each accession and then dehydrated for 72 h at 60 °C by using a modified CTAB method (Elias *et al.*, 2004). Fifty milligrams of ground powder were transferred to a 1.5 mL micro-tube containing 800 μL of CTAB extraction buffer [30 mM EDTA pH 8.0, 0.1 M Tris-HCL pH 8.0, 1.2 M NaCl, 3% CTAB, plus 1% 2-mercaptoethanol added just before use]. After incubation at 65 °C for 1 h, 500 μL of chloroform-isoamylalcohol (24:1) were added and the mixture subsequently centrifuged at 8,000 rpm for 10 min. This step was repeated once again. The supernatant (400 μL) was then transferred to a fresh tube with 350 μL of -20 °C isopropanol and stored at -4 °C for 1 h, whereupon it was once more centrifuged at 8,000 rpm for 10 min. After drying, 200 μL of TE buffer (10 mM Tris-HCl pH 8.0, 1 mM EDTA) and 4 μL RNAse (10 mg/mL) were added to each tube. The DNA was quantified in 4% polyacrylamide gel electrophoresis by using the silver nitrate staining technique (Bassam *et al.*, 1991), so as to visualize the DNA bands in the gels.

### Microsatellite amplification

The amplification reaction was run on a total volume of 10.2 μL, consisting of 0.2 μL of Taq Polymerase (5 U/μL); 1.0 μL 10x Buffer ; 1.0 μL MgCl_2_ (50 mM); 0.5 μL of each primer (F/R) (5pmoles/μL); 1.0 μL dNTP (2.5 mM of each deoxyribonucleotide); 3.0 μL Milli-Q H_2_O and 3.0 μL DNA (5 ng). Based on previous studies (Mühlen *et al.*, 2000; Elias *et al.*, 2004; Peroni *et al.*, 2007), nine microsatellite primers were used (Chavarriaga-Aguirre *et al.*, 1998) (Table 2). PCR reactions were performed with a MWG-BIOTECH Primus 96 thermocycler, with the following sequences: 4 min at 95 °C, 29 cycles of 1 min at 95 °C; 2 min at the annealing temperature defined for each primer (Table 2); 2 min at 72 °C; and a final extension stage of 1 min at 72 °C. Separation of the amplified product was accomplished in 6% polyacrylamide gel electrophoresis at 60 V for 30 min and 120 V for 3 h and 30 min. The gels were stained with silver nitrate (Bassam *et al.*, 1991) and photo-documented.

### Statistical analyses

Genetic diversity parameters, such as the number of alleles per locus, allelic frequency, percent of polymorphic loci, observed average heterozigosity and gene diversity (expected heterozygosity) obtained per locus and per group of accessions, were estimated using GDA software (Lewis and Zaykin, 2000). Allelic frequencies and Nei (1973) genetic diversity parameters were estimated with FSTAT software (Goudet, 2001).

Cluster analysis with the 42 landraces was performed with NTSYS software, by using binary data whereby alleles were transformed into the presence or absence of an SSR band, as well as with the Jaccard similarity coefficient and UPGMA (Unweighted Pair Group Method with an Arithmetic Mean) method. A principal component analysis with binary data was also carried out using the SAS (1999) program, BioStat 4.0 software (Ayres *et al.*, 2005) providing a scatter plot.

## Results

A total of 46 alleles were amplified with nine SSR loci analyzed in the 42 landraces, the number of alleles observed per locus varying from 3 to 6 alleles (Table 2). The number of alleles per polymorphic locus in the five cassava groups varied from 2.1 to 3.8, with an average of 3.3 (Table 3). Specific alleles were detected in the MS, MT and SP groups. Some of these alleles were considered to be rare (0.050 frequency), such as alleles 3 and 5 for GA-5 in the MS group and allele 3 for GA-126 in that of MT.

The groups MS, AM and MT revealed 100% polymorphism, while those of MG and SP presented 88.8%. The observed heterozygosity, 0.265 on an average, varied from 0.233 to 0.288, while higher values, varying from 0.427 to 0.677, were estimated for gene diversity, with an average of 0.570.

According to Nei diversity indices (Nei, 1973), high total diversity (*H*_*T*_ = 0.635) was observed in all the 42 cassava landraces, thus confirming the high variability which can be found in this cross-pollinated and vegetatively propagated crop. However, most of this SSR variability was concentrated within ethno-variety groups (*H*_*S*_ = 0.552), while lower values were due to the proportion of diversity distributed among the groups themselves (*G*_*ST*_*'* = 0.131) (Table 4).

The dendrogram in Figure 2 shows the high genetic variability of landraces, this varying from 0.19 to 0.83 in the Jaccard similarity coefficient. Five groups were defined through cluster analysis. Landraces from all the five regions were classified in the first of these, the five accessions from Frutal (MG) being gathered into a small sub-group of this larger one. As to clustering in the other four groups, landraces only from General Carneiro (MT) were classified in the second group, four from Sonora and Pedro Gomes (MS), and two, one from Cananéia and the other from Ilha Comprida (SP) in the third, two from the Amazon region plus one from Inocência (MS) in the fourth and finally, only one single specimen from Cassilândia (MS) in the fifth.

Similar results were observed through principal component analysis when examining the distribution of the five groups in the four quadrants of a scatter plot compounded with the two principal components, and which explained 25.8% of total variation (data not shown), besides indicating the high genetic variability of the material.

## Discussion

In our study, high genetic diversity was detected in all the five regions in Brazil, with an average of 5.0 alleles per locus, which is in agreement to similar studies with cassava (Muhlen *et al.*, 2000; Faraldo *et al.*, 2000; Fregene *et al.*, 2003; Mkumbira *et al.*, 2003; Elias *et al.*, 2004; Lokko *et al.*, 2006). Peroni *et al.* (2007) evaluated 137 cassava specimens representing 58 landraces from Brazil, by using nine SSR loci, thereby reporting an average of 4.56 alleles per locus, this varying from 2 to 7 alleles. When analyzing 283 accessions from various countries with 67 SSR loci, Fregene *et al.* (2003) found an average of 5.02 alleles per locus for Brazilian landraces.

High gene diversity values of 0.570 on an average were also encountered in our study. By using SSR markers, gene diversity was found to be high in all the cluster groups of cassava analyzed by Lokko *et al.* (2006), with an average of 0.447. When assessing 283 accessions of cassava landraces from Africa and the Neotropics, Fregene *et al.* (2003) also came upon high gene diversity, 0.535 on an average with 67 SSR loci. On the other hand, on studying 137 cassava plants from 58 landraces in Brazil, Peroni (Peroni N, PhD Thesis, UNICAMP, 2004) obtained the even higher value of 0.637 for average expected heterozygosity or gene diversity. All these values are high when compared to the average gene diversity for outcrossing species of 0.205, estimated for all plant species, and 0.159 for dicots (Hamrick and Godt, 1997). These high values also substantiate both the cassava outcrossing breeding system, with multi-loci outcrossing rates estimated at 91.5% when using isozyme markers (Silva *et al.*, 2003), as well as its highly heterozygous nature due to its vegetative mode of reproduction. In French Guiana, Pujol *et al.* (2005) noted a positive correlationship between plant size and heterozygosity, thus concluding that during weeding farmers tended to eliminate the less vigorous plantlets, which in itself could explain the higher levels of heterozygosity reported in the literature.

A larger portion of diversity in this study was found to be concentrated within the groups themselves (*H*_*S*_ = 0.552) on the contrary to group diversity (*G*_*ST*_*'* = 0.131). Faraldo *et al.* (2000), Mühlen *et al.* (2000) and Asante and Offei (2003) also observed that most morphological, isozymatic and molecular variability was concentrated within either the cassava ‘swidden' fields cultivated by traditional farmers or geographic regions. Peroni *et al.* (2007), when using nine SSR loci to analyze 58 cassava landraces, arrived at similar results, thereby explaining that each farmer maintains an appreciable representation of total diversity in his homestead, this diversity not being different from that of other farmers in the same region, and is due to material exchange between relatives and neighbors. Lokko *et al.* (2006) also found that most gene diversity assessed with SSR markers was concentrated within cluster groups of cassava from Africa. The same pattern was observed with sweet potato landraces from the Vale do Ribeira, with greater genetic variability within swidden fields for both morphological (Veasey *et al.*, 2007) and SSR markers (Veasey *et al.*, 2008).

In addition to measuring genetic diversity, one of the objectives in our study was to verify how landraces originating from five different regions in Brazil were mutually related. Results showed a greater proximity of landraces from the states of São Paulo, Mato Grosso do Sul and Amazonas. Faraldo *et al.* (2000), on studying cassava landraces from three distinct groups (the Indigenous Park of Xingu, the Vale do Ribeira in São Paulo and the Amazon region), found greater likeness among landraces from São Paulo and the Amazon. Peroni *et al.* (2007) also identified greater genetic similarity among landraces from the Vale do Ribeira and those of the Rio Negro (Amazon). According to these authors, the samples from São Paulo, represented here by landraces from the Vale do Ribeira, may represent a “historical sample” of Amazonian cassava diversity.

The landraces from Minas Gerais were clustered into a sub-group in the dendrogram (Figure 2), within a larger group of landraces from São Paulo, Mato Grosso do Sul and Amazonas. The landraces collected in Frutal, Minas Gerais, an area dominated by soybeans, pineapples, sugar and pasture dedicated to milk and beef production, were all cultivated in home-gardens and not in ‘swidden' fields. These are known as sweet-varieties and are used for home-cooking. The origin of these landraces is apparently local, with plant material being exchanged among relatives, friends and neighbors and, according to the villagers, have been under cultivation in this area over a long period (Angelo and Amorozo, 2006).

The most differentiated landraces were those from General Carneiro, Mato Grosso. In Mato Grosso, traditional farming is undertaken by local populations, particularly by indians, “quilombolas” and “pantaneiros”. After the 50's more recent settlers began coming from diverse regions of the country, mainly from the south, northeast, Goiás and Minas Gerais (Amorozo, 2000). General Carneiro is an important area for settlement, the high genetic variability of cassava landraces being a consequence of the introduction of genetic material from the settler's place of origin. This municipality presents certain peculiarities that may help to explain the differentiation of local landraces from those of other regions. More extensive modern farming with soybean and cotton crops, predominant in other parts of the state, is a reality for farmers in this area. Information on modern cassava varieties and released by plant breeding institutes in Brazil (Instituto Agronômico - IAC, Embrapa) can easily reach local farmers through the radio and satellite television. However, due to economical and transport limitations, access to this material is very rare. Some local farmers reported impediments in obtaining new varieties for homestead planting, even of those from the same area, due to the difficulty and high cost in transportation. Under local conditions, distances as short as 20 kilometers can constitute an isolation factor.

Both sweet and bitter varieties of cassava are grown by farmers in the States of São Paulo (Amorozo, 2000) and Mato Grosso do Sul, substantial diversity of sweet varieties being encountered in the latter (Zatarin M and Valle TL, personal communication). Sweet varieties have a low level of cyanogenic glucoside content in their roots, whereas the bitter type presents more than 100 ppm fresh weight of cyanide therein which must be detoxified before consumption (Valle *et al.*, 2004). The Vale do Ribeira region, represented by Cananéia, Ilha Comprida and Eldorado, is an area with a high diversity of species and varieties, especially cassava, and it is possible that Tupi-Guarani populations were the main disseminators of both cultivation techniques as well as of the species and varieties to be found there (Peroni N, PhD Thesis, UNICAMP, 2004). It is possible that the greater similarity among landraces from São Paulo and Mato Grosso do Sul can be traced back to migrating populations coming from southern Amazonia, bringing varieties which were introduced into the Vale do Ribeira together with their traditional cultivation techniques (Schaden, 1974). Some cassava varieties were probably carried along with Tupi-Guarani indians migrating from São Paulo to communities in Mato Grosso do Sul (Ladeira MI, PhD Thesis, USP, 2001). Thus, these could be some of the factors contributing to an explanation of the proximity of landraces from São Paulo with those collected in the cerrado ecosystem of Mato Grosso do Sul and the tropical Amazon Forest.

In this study we intended to contribute to a better knowledge of cassava genetic diversity and distribution within and among different regions in Brazil, and promote *in situ* conservation by traditional farmers, also known as *on farm in situ* conservation, of an important genetic and autochthon resource.

## Figures and Tables

**Table 1:** List of the cassava (*Manihot esculenta*) landraces and groups (MG - Minas Gerais; SP - São Paulo; MS - Mato Grosso do Sul; AM - Amazonas; MT - Mato Grosso) used in this study and their respective origins, folk names and classification according to usage.

Code	Municipality (community), State	Folk name	Classification
Group MG			
MG1	Frutal (Aparecida de Minas), MG	-^1^	Sweet
MG2	Frutal (Aparecida de Minas), MG	-	Sweet
MG3	Frutal (Boa Esperança), MG	-	Sweet
MG4	Frutal (Boa Esperança), MG	-	Sweet
MG5	Frutal (Aparecida de Minas), MG	-	Sweet
Group SP			
SP1	Eldorado, SP	Mandioca roxa	-
SP2	Cananéia (Agrossolar), SP	Aipim 5 min	Sweet
SP3	Cananéia (Rio Branco), SP	Mandioca amarela	Sweet
SP4	Cananéia (Rio Branco), SP	Mandioca roxa	Sweet
SP5	Cananéia (Rio Branco), SP	Mandioca branca	Sweet
SP6	Cananéia (Porto Cubatão), SP	Mandioca manteira	Sweet
SP7	Ilha Comprida (Pedrinhas), SP	Branca	Bitter
Group MS			
MS1	Sonora, MS	Vassourinha	Bitter
MS2	Sonora, MS	Macaxeira	Sweet
MS3	Pedro Gomes, MS	-	-
MS4	Pedro Gomes, MS	Amarela	-
MS5	Rio Verde de MT, MS	Amarela manteiga	-
MS6	Rio Verde de MT, MS	Macaxeira	Sweet
MS7	Costa Rica, MS	-	-
MS8	Cassilândia, MS	Amarela	-
MS9	Paranaíba, MS	-	-
MS10	Inocência, MS	Vassoura amarela	-
Group AM			
AM 1	Uarini (Aiucá), AM	Pelonha	Bitter
AM 2	Maraã (Monte Sinai), AM	Tambaqui	Sweet
AM 3	Maraã, (Boa Esperança), AM	Leônico	Bitter
AM 4	Maraã (Boa Esperança), AM	Caboclinha	Sweet
AM 5	Maraã (S. Paulo do Coraci), AM	Tartarugão	Bitter
AM 6	Maraã (Calafate), AM	Catombo	Bitter
AM 7	Uarini (Aiucá), AM	Valdivina	Bitter
AM 8	Alvarães (Jarauá), AM	Brasileirinha	Sweet
AM 9	Maraã (Nova Samaria), AM	Bodozinho	Bitter
AM 10	Uarini (Marirana), AM	Sem nome	Bitter
Group MT			
MT1	G. Carneiro, Household 1, MT	Da praia	-
MT2	G. Carneiro, Household 2, MT	Castelão	Sweet
MT3	G. Carneiro, Household 2, MT	Pracati	Bitter
MT4	G. Carneiro, Household 3, MT	Cenoura	Sweet
MT5	G. Carneiro, Household 4, MT	Cacau	Sweet
MT6	G. Carneiro, Household 4, MT	Menina	Sweet
MT7	G. Carneiro, Household 5, MT	Mucuruna	Bitter
MT8	G.Carneiro, Household 5, MT	Matrinxã	Sweet
MT9	G. Carneiro, Household 3, MT	Engana vizinho	Sweet
MT10	G. Carneiro, Household 4, MT	Paraguainha	Bitter

^1^Unknown.

**Table 2:** Primer sequences (forward/reserve) used in SSR analyses and their respective size- range (bp)^1^, annealing temperature (*T*_*a*_), number of alleles per locus (*A*), observed heterozygosity ( ${\bar{H}}_{0}$) and expected heterozygosity ( ${\bar{H}}_{e}$).

Microsatellite name^1^	5' to 3' Primer sequence	Size-range (bp)^2^	*T*_*a*_ (°C)	*A*	${\bar{H}}_{0}$	${\bar{H}}_{e}$
GA-5	TAATGTCATCGTCGGCTTCG GCTGATAGCACAGAACACAG	120-130	60	5	0.405	0.459
GA-12	GATTCCTCTAGCAGTTAAGC CGATGATGCTCTTCGGAGGG	140-150	57	5	0.167	0.752
GA-21	GGCTTCATCATGGAAAAACC CAATGCTTTACGGAAGAGCC	110-120	62	3	0.190	0.615
GA-126	AGTGGAAATAAGCCATGTGATG CCCATAATTGATGCCAGGTT	170-210	57	6	0.500	0.784
GA-127	CTCTAGCTATGGATTAGATCT GTAGCTTCGAGTCGTGGGAGA	210-235	59	6	0.262	0.773
GA-131	TTCCAGAAAGACTTCCGTTCA CTCAACTACTGCACTGCACTC	95-140	54	5	0.119	0.707
GA-134	ACAATGTCCCAATTGGAGGA ACCATGGATAGAGCTCACCG	295-310	52	5	0.095	0.641
GA-136	CGTTGATAAAGTGGAAAGAGCA ACTCCACTCCCGATGCTCGC	145-165	64	5	0.143	0.746
GA-140	TTCAAGGAAGCCTTCAGCTC GAGCCACATCTACTCGACACC	150-165	62	5	0.476	0.684
Mean				5	0.262	0.684

^1^Chavarriaga-Aguirre *et al.* (1998). ^2^Values obtained in this study, similar to those of Chavarriaga-Aguirre *et al.* (1998).

**Table 3:** Number of individuals analyzed (*N*), mean number of alleles per polymorphic locus ( $\bar{A}$), percentage of polymorphic loci (*P*), mean observed heterozygosity ( ${\bar{H}}_{0}$) and gene diversity ( ${\bar{H}}_{e}$) for five groups of cassava: MG - Minas Gerais; SP - São Paulo; MS - Mato Grosso do Sul; AM - Amazonas; MT - Mato Grosso.

				Mean heterozygosity	
Groups	*N*	$\bar{A}$	*P* (%)	${\bar{H}}_{0}$	${\bar{H}}_{e}$
MG	5	2.11	88.88	0.288	0.427
SP	7	3.66	88.88	0.269	0.610
MS	10	3.77	100.00	0.255	0.677
AM	10	3.44	100.00	0.233	0.588
MT	10	3.44	100.00	0.277	0.550
Mean	8.40	3.28	95.55	0.265	0.570

**Table 4:** Nei (1973) genetic diversity parameters^1^ for each locus and for the total evaluated loci considering five groups of cassava: MG - Minas Gerais; SP - São Paulo; MS - Mato Grosso do Sul; AM - Amazonas; MT - Mato Grosso.

Loci	*H*_*S*_	*H*_*T*_	*D*_*ST*_	*G*_*ST*_
GA-5	0.450	0.453	0.003	0.006
GA-12	0.418	0.618	0.200	0.324
GA-21	0.316	0.574	0.258	0.450
GA-126	0.679	0.711	0.032	0.045
GA-127	0.632	0.737	0.105	0.142
GA-131	0.556	0.610	0.054	0.089
GA-134	0.577	0.616	0.039	0.063
GA-136	0.706	0.730	0.025	0.034
GA-140	0.637	0.668	0.031	0.047

Total	0.552	0.635	0.083	0.131

^1^*H*_*S*_ (within-groups diversity component), *H*_*T*_ (total species-diversity), *D*_*ST*_ (between-groups diversity component), *G*_*ST*_ (proportion of genetic diversity attributed to the between-groups component), where *G*_*ST*_ = *D*_*ST*_/*H*_*T.*_

## References

[Amorozo2000] Amorozo M.C.M. (2000). Management and conservation of *Manihot esculenta* Crantz germplasm by traditional farmers in Santo Antonio do Leverger, Mato Grosso State, Brazil. Etnoecologica.

[AngeloandAmorozo2006] Angelo G.A., Amorozo M.C.M., Albuquerque U.P., Marins J.F.A., Almeida C.F.C.B.R. (2006). Diversidade de tubérculos alimentícios em povoados rurais no Município de Frutal, Minas Gerais, Brasil. Tópicos em Conservação e Etnobotânica de Plantas Alimentícias.

[AsanteandOffei2003] Asante I.K., Offei S.K. (2003). RAPD-based genetic diversity study of fifty cassava (*Manihot esculenta* Crantz) genotypes. Euphytica.

[Ayresetal2005] Ayres M., Ayres M., Ayres D.L., Santos A.A.S. (2005). BioEstat 4.0: Aplicações estatísticas nas áreas das ciências biológicas e médicas.

[Bassametal1991] Bassam B.J., Caetano-Anolles G., Gresshoff P.M. (1991). Fast and sensitive silver staining of DNA in polyacrylamide gels. Anal Biochem.

[Brown1978] Brown A.H.D. (1978). Isozymes, plant population genetic structure and genetic conservation. Theor Appl Genet.

[Cabraletal2002] Cabral B.L.R., Souza J.A.B., Ando A., Veasey E.A., Cardoso E.R. (2002). Isoenzymatic variability of cassava accessions from different regions in Brazil. Sci Agric.

[CarvalhoandSchaal2001] Carvalho L.J.C.B., Schaal B.A. (2001). Assessing genetic diversity in the cassava (*Manihot esculenta* Crantz) germplasm collection in Brazil using PCR-based markers. Euphytica.

[Chavarriaga-Aguirreetal1998] Chavarriaga-Aguirre P., Maya M.M., Bonierbale M.W., Kresovich S., Fregene M.A., Tohme J., Kochert G. (1998). Microsatellites in cassava (*Manihot esculenta* Crantz): Discovery, inheritance and variability. Theor Appl Genet.

[Colomboetal1998] Colombo C., Second G., Valle T.L., Charrier A. (1998). Genetic diversity characterization of cassava cultivars (*Manihot esculenta* Crantz). I. RAPD markers. Genet Mol Biol.

[Cresteetal2003] Creste S., Neto A.T., Silva S.O., Figueira A. (2003). Genetic characterization of banana cultivars (*Musa* spp.) from Brazil using microsatellite markers. Euphytica.

[Eliasetal2000] Elias M., Panaud O., Robert T. (2000). Assessment of genetic variability in a traditional cassava (*Manihot esculenta* Crantz) farming system, using AFLP markers. Heredity.

[Eliasetal2004] Elias M., Mühlen G.S., McKey D., Roa A.C., Tohme J. (2004). Genetic diversity of traditional South American landraces of cassava (*Manihot esculenta* Crantz): An analysis using microsatellites. Econ Bot.

[Emperaireetal2001] Emperaire L., Pinton F., Second G. (2001). Dinámica y manejo de la diversidad de las variedades de yuca del noroccidente amazónico (Brasil). Etnoecológica.

[Faraldoetal2000] Faraldo M.I.F., Silva M.R., Ando A., Martins P.S. (2000). Variabilidade genética de etnovariedades de mandioca em regiões geográficas do Brasil. Sci Agric.

[Fregeneetal2003] Fregene M., Suarez M., Mkumbira J., Kulembeka H., Ndedya E., Kulaya A., Mitchel S., Gullberg U., Rosling H., Dixon A. (2003). Simple sequence repeat marker diversity in cassava landraces: Genetic diversity and differentiation in an asexually propagated crop. Theor Appl Genet.

[Goudet2001] Goudet J. (2001). FSTAT, v. 1.2: A computer program to calculate F-statistics. Heredity.

[HamrickandGodt1997] Hamrick J.L., Godt M.J.W. (1997). Allozyme diversity in cultivated crop. Crop Sci.

[Jianchuetal2001] Jianchu X., Yongping Y., Yingdong P., Ayad W.G., Eyzaguirre P.B. (2001). Genetic diversity in taro (*Colocasia esculenta* Schott, Araceae) in China: An ethnobotanical and genetic approach. Econ Bot.

[Lokkoetal2006] Lokko Y., Dixon A., Offei S., Danquah E., Fregene M. (2006). Assessment of genetic diversity among African cassava *Manihot esculenta* Crantz accessions resistant to the cassava mosaic virus disease using SSR markers. Genet Resour Crop Evol.

[Louetteetal1997] Louette D., Charrier A., Berthaud J. (1997). *In situ* conservation of maize in Mexico, genetic diversity and maize seed management in a traditional community. Econ Bot.

[Malapaetal2005] Malapa R., Arnau G., Noyer J.L., Lebot V. (2005). Genetic diversity of the greater yam (*Dioscorea alata* L.) and relatedness to *D. nummularia* Lam. and *D. transversa* Br. as revealed with AFLP markers. Genet Resour Crop Evol.

[Martins1994] Martins P.S. (1994). Biodiversity and agriculture: Patterns of domestication of Brazilian native plants species. An Acad Bras Cienc.

[Martins2001] Martins P.S., Vieira I.C.G., Silva J.M.C., Oren D.C., D'Incao M.A. (2001). Dinâmica evolutiva em roças de caboclos amazônicos. Diversidade Biológica e Cultura da Amazônia.

[Mkumbiraetal2003] Mkumbira J., Chiwona-Karltun L., Langercrantz U., Mahungu N., Saka J., Mhone A., Bokanga M., Brimer L., Gullberg U., Rosling H. (2003). Classification of cassava into ‘bitter' and ‘cool' in Malawi: From farmers' perception to characterization by molecular markers. Euphytica.

[Muhlenetal2000] Mühlen G.S., Martins P.S., Ando A. (2000). Variabilidade genética de etnovariedades de mandioca, avaliada por marcadores de DNA. Sci Agric.

[Nei1973] Nei M. (1973). Analysis of gene diversity in subdivided populations. Proc Natl Acad Sci USA.

[Peroni2007] Peroni N., Boef W.S., Thijssen M.H., Ogliari J.B., Sthapit B.R. (2007). Manejo e domesticação de mandioca por caiçaras da Mata Atlântica e ribeirinhos da Amazônia. Biodiversidade e Agricultores: Fortalecendo o Manejo Comunitário.

[PeroniandMartins2000] Peroni N., Martins P.S. (2000). Influência da dinâmica agrícola itinerante na geração de diversidade de etnovariedades cultivadas vegetativamente. Interciencia.

[Peronietal2007] Peroni N., Kageyama P.Y., Begossi A. (2007). Molecular differentiation, diversity, and folk classification of ‘‘sweet'' and ‘‘bitter'' cassava (*Manihot esculenta*) in Caiçara and Caboclo management systems (Brazil). Genet Resour Crop Evol.

[Pujoletal2005] Pujol B., Gigot G., Laurent G., Pinheiro-Kluppel M., Elias M., McKey H.M., McKey D. (2005). Germination ecology of cassava (*Manihot esculenta* Crantz, Euphorbiaceae) in traditional agroecosystems: Seed and seedling biology of a vegetatively propagated domesticated plant. Econ Bot.

[Resendeetal2004] Resende A.G., Filho P.S.V., Machado M.F.P.S. (2004). Esterase polymorphism marking cultivars of *Manihot esculenta*, Crantz. Braz Arch Biol Technol.

[Sambattietal2000] Sambatti J.B.M., Martins P.S., Ando A. (2000). Distribuição da diversidade isoenzimática e morfológica da mandioca na agricultura autóctone de Ubatuba. Sci Agric.

[Sambattietal2001] Sambatti J.B.M., Martins P.S., Ando A. (2001). Folk taxonomy and evolutionary dynamics of cassava: A case study in Ubatuba, Brazil. Econ Bot.

[SAS1999] SAS (1999). The SAS System for Windows. Software, Release 8.2 TS Level02MO.

[Schaden1974] Schaden E. (1974). Aspectos Fundamentais da Cultura Guarani.

[Silvaetal2003] Silva R.M., Bandel G., Martins P.S. (2003). Mating system in an experimental garden composed of cassava (*Manihot esculenta* Crantz) ethnovarieties. Euphytica.

[Valleetal2004] Valle T.L., Carvalho C.R.L., Ramos M.T.B., Mühlen G.S., Villela O.V. (2004). Conteúdo cianogênico em progênies de mandioca originadas do cruzamento de variedades mansas e bravas. Bragantia.

[Veaseyetal2007] Veasey E.A., Silva J.R.Q., Rosa M.S., Borges A., Bressan E.A., Peroni N. (2007). Phenology and morphological diversity of sweet potato (*Ipomoea batatas*) landraces of the Vale do Ribeira. Sci Agric.

[Veaseyetal2008] Veasey E.A., Borges A., Rosa M.S., Queiroz-Silva J.R., Bressan E.A., Peroni N. (2008). Genetic diversity assessed with microsatellites in Brazilian sweetpotato (*Ipomoea batatas* (L.) Lam) landraces. Genet Mol Biol.

[Zacariasetal2004] Zacarias A.M., Botha A.M., Labuschagne M.T., Benesi I.R.M. (2004). Characterization and genetic distance analysis of cassava (*Manihot esculenta* Crantz) germplasm from Mozambique using RAPD fingerprinting. Euphytica.

